# Efficacy of PD-1/PD-L1 inhibitors in ovarian cancer: a single-arm meta-analysis

**DOI:** 10.1186/s13048-021-00862-5

**Published:** 2021-08-28

**Authors:** Jue Zhu, Lifeng Yan, Qiming Wang

**Affiliations:** Department of Gynecology, Ningbo Women & Children’s Hospital, Ningbo, 315211 China

**Keywords:** PD-1/PD-L1 inhibitors, Immunotherapy, Ovarian cancer

## Abstract

Several studies have evaluated the efficacy of PD-1/PD-L1 inhibitors in ovarian cancer; however, the response rate varies. This study aims to explore the efficacy of anti-PD-1/PD-L1 therapy in ovarian cancer. A quantitative meta-analysis was performed through a systematic search in PubMed, Web of Science, and the Cochrane Library. The pooled ORR was calculated and compared. Fifteen trials were included in this meta-analysis. Our analyses showed that the pooled ORR of all included studies was 19% (95% CI: 13%, 27%). Single PD-1/PD-L1 inhibitors had the lowest ORR of 9% (95% CI: 7%, 12%), while the combination of PD-1/PD-L1 inhibitors and chemotherapy had the highest ORR of 36% (95% CI: 24%, 51%). This study showed that PD-1/PD-L1 inhibitors alone have limited efficacy for ovarian cancer. The combination of PD-1/PD-L1 inhibitors and chemotherapy could be chosen as the recommended modality for further study.

## Background

Ovarian cancer (OC) is the eighth most common cancer in women worldwide (tenth in China) [1]. Due to its insidious onset and vague presenting symptoms, almost two-thirds of patients are diagnosed with advanced disease [2], which is associated with significant mortality. The 5-year survival rate ranges from 35% to 45.6% in patients with advanced-stage disease [3]. Platinum/taxane-based chemotherapy with or without bevacizumab is still the standard of care for advanced OC. Currently, the overall response rate (ORR) of primary treatment is 60–80%; however, 70% of patients relapse within 5 years, and many of them develop drug-resistant disease [4]. Poly ADP-ribose polymerase (PARP) inhibitors are shifting the paradigm of care for OC patients. Nevertheless, new strategies are still needed for these patients.

Immune checkpoint inhibitor therapies have transformed cancer treatment in various solid malignant tumors, such as melanoma, non-small cell lung cancer, liver cancer, and renal cell carcinomas. In particular, anti-PD-1 or PD-L1 therapy is becoming increasingly popular in cancer therapy. In contrast to traditional chemotherapy or targeted therapy, immunotherapy shows a clear plateau in the overall survival curve, representing long-term survivors. Currently, a series of phase I/II studies have evaluated the efficacy of anti-PD-1 or PD-L1 therapy in OC, with ORR ranging from 8 to 60% and median progression-free survival (PFS) times ranging from 2 to 10 months [5–7]. The quite different responses to anti-PD-1 or PD-L1 therapy might be attributed to different combination therapies or OC types. Therefore, it is necessary to investigate specific combination therapies or subtypes of OC that benefit most from immunotherapy. Most of these trials were designed as single-arm trials and had noncomparable forms. Therefore, we conducted this quantitative meta-analysis to explore the efficacy of anti-PD-1/PD-L1 therapy in OC.

## Methods

### Search strategy

We searched PubMed, Web of Science, and the Cochrane Library from 1966 to January 19, 2021. We also reviewed records of the American Society of Clinical Oncology (ASCO) and the European Society for Medical Oncology (ESMO). The following search terms were used: “PD-L1”, “PD-1”, “pembrolizumab”, “nivolumab”, “atezolizumab”, “durvalumab”, “avelumab”, and “ovarian cancer”. The references of literature reviews and original articles were also scanned to avoid missing any qualified studies.

### Inclusion and exclusion criteria

The inclusion criteria were as follows: (1) prospective clinical studies (including randomized control trials and single-arm studies); (2) articles investigating PD-1/PD-L1 inhibitors in OC patients; and (3) studies reporting the overall response rate (ORR). The exclusion criteria were as follows: (1) article type: letters, editorials, expert opinions, case reports and reviews; (2) studies without usable data; and (3) duplicate publications.

### Data extraction

Two investigators extracted data from the eligible studies independently, and any disagreements were resolved by discussion with a third investigator. For each study, the following characteristic information was recorded: first author, year of publication, number of patients, ORR, disease control rate (DCR), therapeutic regimen, and response to previous platinum-based regimen.

### Quality assessment

Most of the included studies were single arm or non-controlled studies. Therefore, the Newcastle–Ottawa Scale (NOS) tools were used to assess the quality of included studies [8]. Studies with more than 4 stars were included for further analysis. Any discrepancies were resolved by consensus.

### Statistical analysis

Statistical analyses of the pooled ORR were performed using R version 3.5.2. The heterogeneity of the data was evaluated by chi-square Q test and I^2^ statistic. For the Q test, a p value less than 0.05 indicated significant heterogeneity; for the I^2^ statistics, an I^2^ value greater than 50% was considered significant heterogeneity. Meta-regression and subgroup analyses were performed to identify the factor contributing risk of bias.

## Results

### Patient characteristics

**Fig. 1:**
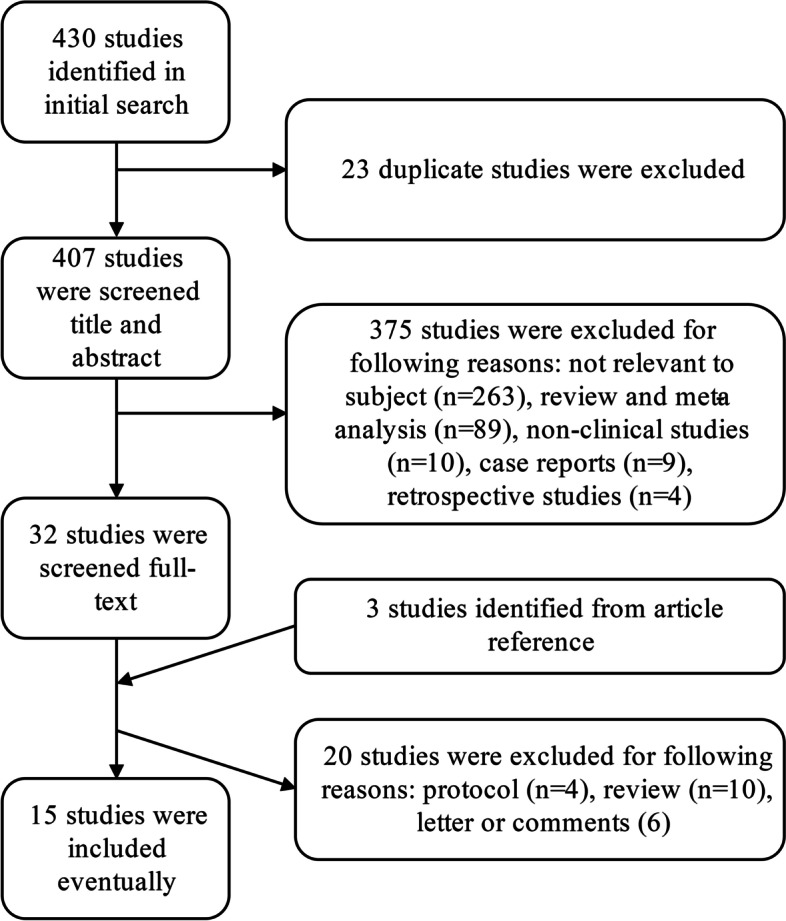
The flow diagram of this meta-analysis

The initial search yielded 430 records. After screening the titles and abstracts, 35 full-text articles were assessed for eligibility. Finally, a total of 15 articles were included in this study [5–7, 9–20]. The study selection process is shown in Fig. 1. Among these 15 studies, the administered PD-1/PD-L1 inhibitors were pembrolizumab (6), nivolumab (3), durvalumab (3), atezolizumab (2), and avelumab (1). Thirteen of 15 studies were assessed as 7–9 stars, and 2 were assessed as 5 stars. The details are summarized in Table 1.

**Table 1:** Characteristics of 15 included studies

First Author	Year	Sample Size	Interventions	Platinum-resistant	NOS score
Hamanishi [9]	2015	20	Nivolumab	Yes	8
Disis [10]	2019	125	Avelumab	NA	8
Konstantinopoulos [11]	2019	60	Pembrolizumab + niraparib	Yes	8
Liu [12]	2019	9	Atezolizumab	NA	8
Liu [13]	2019	38	Nivolumab + bevacizumab	NA	8
Matulonis [5]	2019	376	Pembrolizumab	NA	8
Varga [14]	2019	26	Pembrolizumab	NA	8
Walsh [6]	2019	14	Pembrolizumab + cisplatin + gemcitabine	Yes	5
Lampert [15]	2020	35	Durvaluamb + olaparib	NA	7
Lee [16]	2020	23	Pembrolizumab + pegylated liposomal doxorubicin	Yes	7
Moroney [17]	2020	12	Atezolizumab + bevacizumab	Yes	7
O'Cearbhaill [18]	2020	40	Durvalumab + pegylated liposomal doxorubicin	Yes	5
Zamarin [19]	2020	27	Pembrolizumab + folate receptor alpha vaccine	Yes	9
Zamarin [20]	2020	100	Nivolumab + ipilimumab vs nivolumab	NA	7
Zsiros [7]	2020	40	Pembrolizumab + bevacizumab + cyclophosphamide	NA	8

### Efficacy

**Fig. 2:**
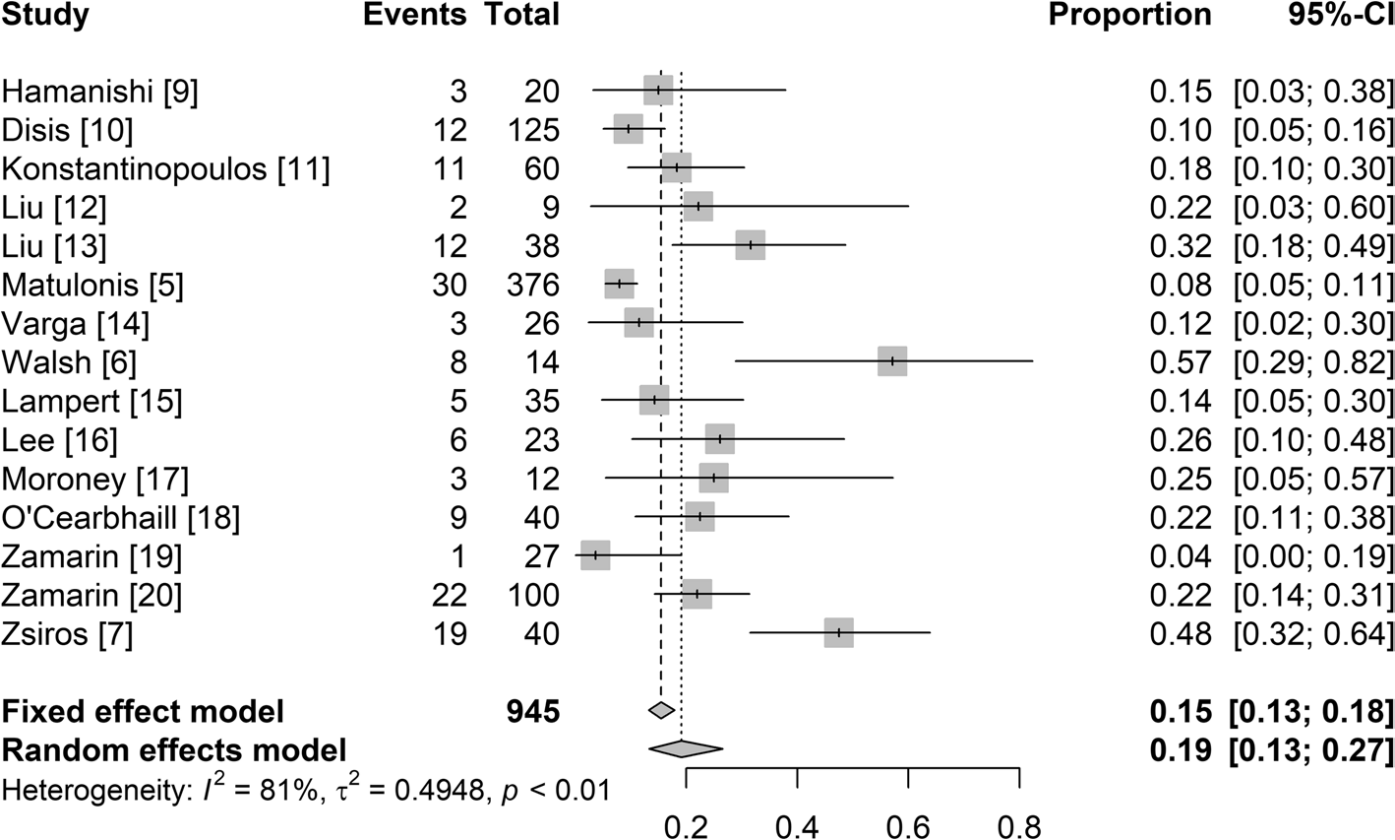
Summary overall response rate for all included studies

All included studies reported the ORR as the clinical activity outcome. The ORRs across the studies varied from 4 to 48%. The random-effects model was adopted because of significant heterogeneity (I^2^ = 81%, *p* < 0.01). The analysis showed a pooled ORR of 19% (95% CI: 13%, 27%) (Fig. 2). As significant heterogeneity in the ORR existed across the studies, meta-regression and subgroup analyses were performed to explore the potential sources of heterogeneity.

### Meta-regression and subgroup analyses

Previous studies showed single PD-1/PD-L1 inhibitors had limited response rate. Additional, platinum-resistant OC had poor response to subsequent therapy. Therefore, regimen combination and platinum-resistant status were included for meta-regression analysis. The results showed immunotherapy regimen combination (single PD-1/PD-L1 inhibitor vs. combination of PD-1/PD-L1 inhibitor with other anti-tumor drugs, *p* < 0.003) contributed to heterogeneity of ORR, while whether platinum-sensitive or -resistance did not influence ORR.

#### ORR in Different PD-1/PD-L1 inhibitor combinations

**Fig. 3:**
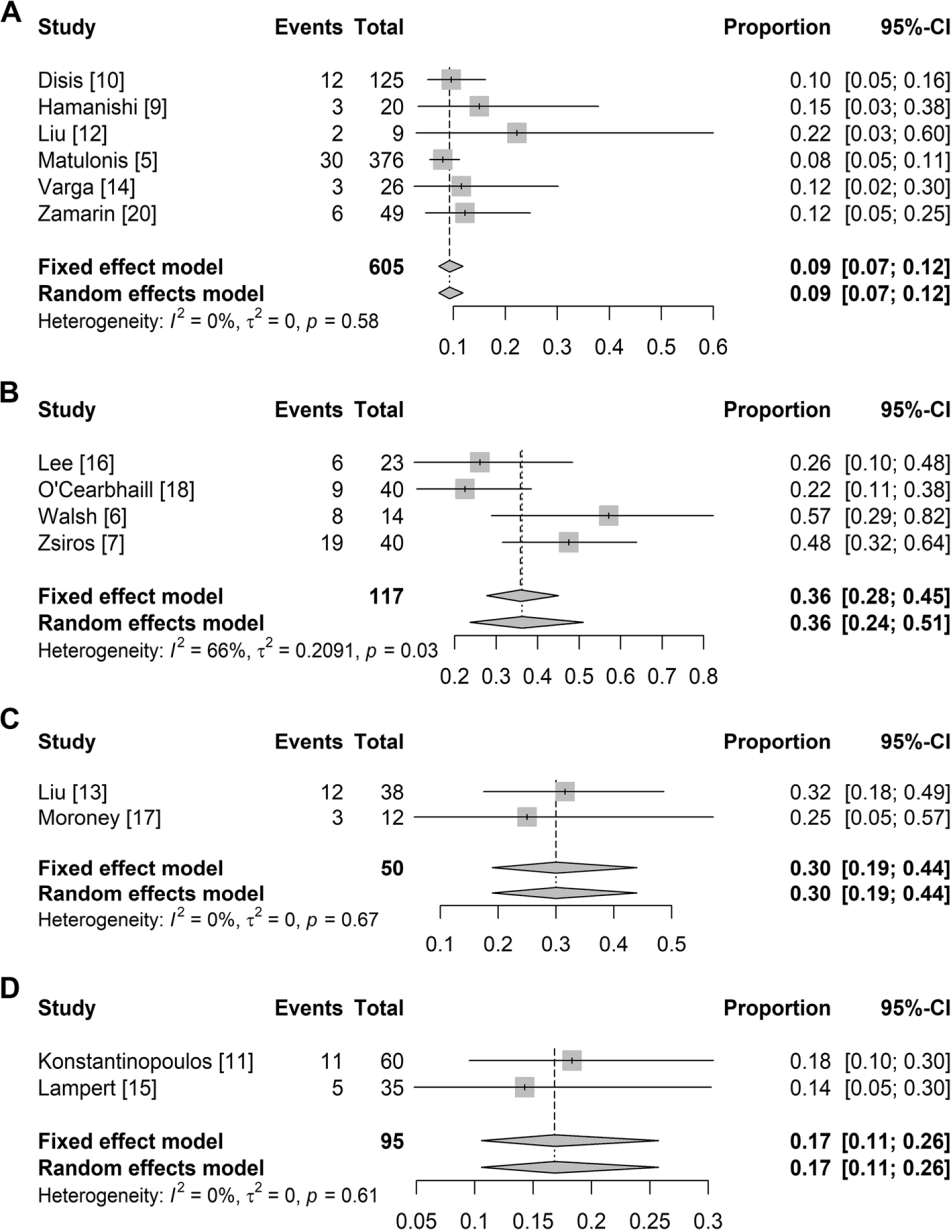
Summary overall response rate for different treatment combination. Summary overall response rate for single PD-1/PD-L1 inhibitors (**A**), PD-1/PD-L1 inhibitors combined with chemotherapy (**B**), PD-1/PD-L1 inhibitors combined with antiangiogenic therapy (**C**), and PD-1/PD-L1 inhibitors combined with PARP inhibitors (**D**)

Six studies on a single PD-1/PD-L1 inhibitor had usable ORR data. The pooled ORR was 9% (95% CI: 7%, 12%) without significant heterogeneity (I^2^ = 0%, *p* = 0.58) (Fig. 3A). Four studies on a combination of PD-1/PD-L1 inhibitors and chemotherapy reported ORR data. The pooled ORR was 36% (95% CI: 24%, 51%), and significant heterogeneity existed (I^2^ = 66%, *p* = 0.03) (Fig. 3B). Two studies on a combination of PD-1/PD-L1 inhibitors and antiangiogenic therapy reported ORR data. The pooled ORR was 30% (95% CI: 19%, 44%) without significant heterogeneity (I^2^ = 0%, *p* = 0.67) (Fig. 3C). Two studies on a combination of PD-1/PD-L1 inhibitors and PARP inhibitors reported ORR data. The pooled ORR was 17% (95% CI: 11%, 26%) without significant heterogeneity (I^2^ = 0%, *p* = 0.61) (Fig. 3D).

#### ORR of platinum-resistant and platinum-sensitive patients

**Fig. 4:**
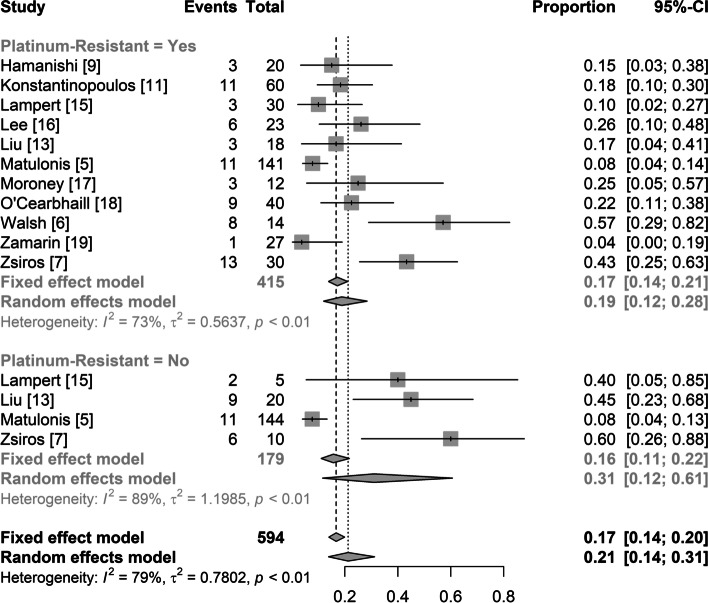
Summary overall response rate for platinum-resistant and platinum-sensitive ovarian cancer

Eleven studies with a total of 594 patients reported the ORR according to prior treatment response (platinum-resistant and platinum-sensitive). The pooled ORR was 21% (95% CI: 14%, 31%) with significant heterogeneity (I^2^ = 79%, *p* < 0.01) (Fig. 4). In 11 studies on platinum-resistant patients, the pooled ORR was 19% (95% CI: 12%, 28%) with significant heterogeneity (I^2^ = 73%, *p* < 0.01). In 4 studies on platinum-sensitive patients, the pooled ORR was 31% (95% CI: 12%, 61%) with significant heterogeneity (I^2^ = 89%, *p* < 0.01) (Fig. 4).

**Fig. 5:**
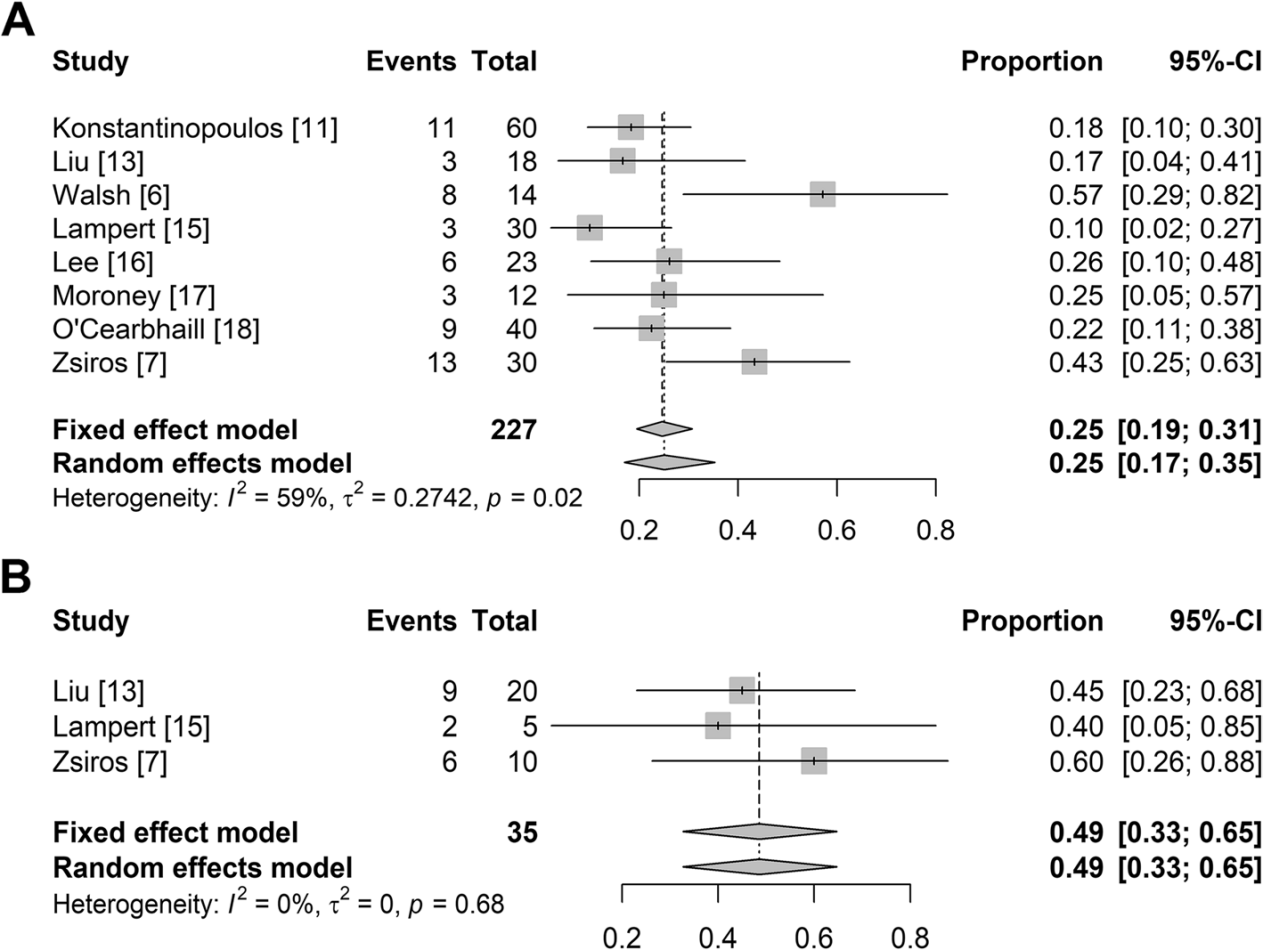
Summary overall response rate for platinum-resistant and platinum-sensitive ovarian cancer excluding treatment arms with a single PD-1/PD-L1 inhibitor

In light of the poor treatment response of single PD-1/PD-L1 inhibitors, we performed meta-analyses separately in platinum-resistant and platinum-sensitive patients, excluding those treatment arms with a single PD-1/PD-L1 inhibitor. In 8 studies on platinum-resistant patients, the pooled ORR was 25% (95% CI: 17%, 35%) with significant heterogeneity (I^2^ = 59%, *p* = 0.02; Fig. 5A). In 3 studies on platinum-sensitive patients, the pooled ORR was 49% (95% CI: 33%, 65%) without significant heterogeneity (I^2^ = 0%, *p* < 0.68; Fig. 5B).

### Publication bias

**Fig. 6:**
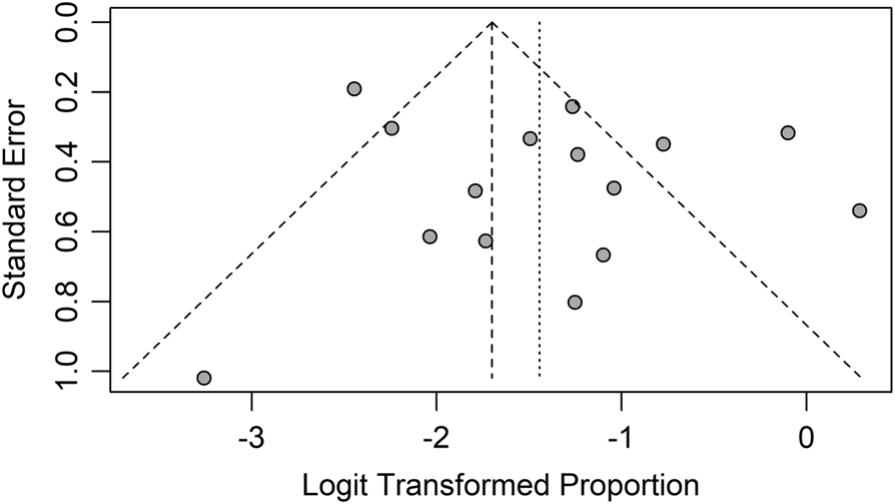
Begg’s funnel plot for overall response rate

The funnel plot for the ORR of the included studies was roughly symmetric (Fig. 6). We also performed Egger’s and Begg’s tests to assess the presence of publication bias in this study. No significantly different results emerged, with *p* = 0.331 for Egger’s test and *p* = 0.656 for Begg’s test.

## Discussion

This study included 15 clinical trials involving 945 patients to evaluate the efficacy of PD-1/PD-L1 inhibitors in treating advanced OC. The pooled results showed that the ORR was 19%. Single PD-1/PD-L1 inhibitors showed limited efficacy, with an ORR of 9%, while combination with chemotherapy showed an increased ORR of 36%. In addition, PD-1/PD-L1 inhibitors had a higher ORR in platinum-sensitive OC than in platinum-resistant OC (31% vs 19%).

Immune checkpoint inhibitors, especially PD-1/PD-L1 inhibitors, are changing the treatment paradigm in certain cancers, such as melanoma and non-small cell lung cancer. The overall ORR with single PD-1/PD-L1 inhibitors across other cancers was approximately 20%, while it was 9% in OC. Previous studies have shown that PD-L1 expression (tumor cells and/or tumor-infiltrating lymphocytes), tumor mutational burden (TMB), microsatellite instability (MSI) and/or mismatch repair (MMR) deficiency are effective predictive biomarkers for anti-PD1/PD-L1 therapy. However, KEYNOTE-028 showed a poor ORR for PD-1 inhibitors, even in PD-L1-positive OC patients [14]. Additionally, KEYNOTE-100 showed a low rate of MSI in OC. As for TMB, it was also very low in OC patients. Therefore, seeking an optimal treatment modality with PD-1/PD-L1 inhibitors seems necessary before identifying a better predictive biomarker.

Vascular endothelial growth factor (VEGF) creates an immunosuppressive microenvironment within cancers by suppressing dendritic cell maturation, increasing the Treg population and stimulating the growth of myeloid-derived suppressor cells in the tumor microenvironment [21, 22]. Bevacizumab can reverse these VEGF-mediated immunosuppressive effects on the tumor microenvironment, potentially augmenting immune-mediated antitumor activity. Several studies have demonstrated the synergistic effect between antiangiogenic agents and PD-1/PD-L1 inhibitors in solid tumors, including renal cancer, non-small lung cancer, and endometrial cancer [23–27]. OC is known to highly express VEGF, which serves as a major driver of tumor neovascularization and local immune suppression [28]. Therefore, anti-VEGF agents could theoretically enhance the efficacy of immunotherapy in OC. This study also showed a high ORR of 30% in OC patients treated with antiangiogenic agents and PD-1/PD-L1 inhibitors.

In recent years, increasing evidence has shown that chemotherapy is not only a cytotoxic agent but also a stimulator of tumor-specific immune responses. Chemotherapy involves the stimulation of anticancer immunity either by initiating the release of immunostimulatory molecules from dying cancer cells or by mediating off-target effects on immune cell populations [29]. On the one hand, chemotherapy could induce immunogenic cell death (ICD), enabling the release of neoantigens and signals to antigen-presenting cells; on the other hand, chemotherapy was found to reduce the number and activity of immune-suppressive cells, including myeloid-derived suppressor cells and Treg cells [30–32]. Therefore, chemotherapy can theoretically initiate or restore anticancer immune responses by converting immunologically “cold” tumors into “hot” tumors. Several studies have shown clinical activities with a combination of immunotherapy and chemotherapy [33, 34]. This study also showed a high ORR of 36% in OC patients treated with chemotherapy and PD-1/PD-L1 inhibitors.

Platinum-resistant OC is a dismal disease and has a low response to subsequent chemotherapy. In this study, we found that the pooled ORR was 25% in studies on platinum-resistant patients and 49% in studies on platinum-sensitive patients. This might be attributed to the immunosuppressive environment in platinum-resistant OC. Data on the tumor microenvironment of platinum-resistant OC showed low CD8 + T cell infiltration and highly activated CD4 + T cells [9, 35].

This study had some limitations. First, most of the included articles were noncomparable studies, and some of them had small sample sizes. Second, the PD-1/PD-L1 inhibitors were different among studies, which inevitably caused bias. Third, the complete data were hardly accessible in some studies to perform subgroup analysis.

## Conclusions

We believe that conducting this meta-analysis was timely and necessary. PD-1/PD-L1 inhibitors alone have limited efficacy for OC. Combination with other therapeutics might be a promising treatment option. The combination of PD-1/PD-L1 inhibitors and chemotherapy showed the highest ORR and could be chosen as the recommended modality for further study.

## Data Availability

The datasets used and/or analyzed during the current study are available from the corresponding author on reasonable request.

## Abbreviations

PM: Peritoneal carcinomatosis
OC: Ovarian cancer
ORR: Overall response rate
PARP: Poly ADP-ribose polymerase
PFS: Progression-free survival
